# Effect of myopia and astigmatism deepening on the corneal biomechanical parameter stress-strain index in individuals of Chinese ethnicity

**DOI:** 10.3389/fbioe.2022.1018653

**Published:** 2022-11-07

**Authors:** Yan Liu, Chenjiu Pang, Shuai Ming, Qi Fan

**Affiliations:** Henan Provincial People’s Hospital, Henan Eye Hospital, Zhengzhou, China

**Keywords:** myopia, astigmatism, corneal biomechanics, stress–strain index, spherical equivalent, intraocular pressure, Corvis ST, Chinese

## Abstract

**Purpose:** To investigate the differences in corneal biomechanical parameter stress–strain index (SSI) among different degrees of myopic eyes in Chinese individuals and to analyze the relevant factors of the SSI.

**Methods:** This study analyzed the right eyes of 240 participants (240 eyes) aged 18–34 years. The participants were divided into low-, moderate-, high-, and ultra-high myopia groups according to their spherical equivalent (SE), with 60 eyes included in each group. Spherical, cylinder, and SE were measured *via* automatically integrated optometry. Intraocular pressure (IOP) was measured using a non-contact tonometer. AL was measured using an IOLMaster device. Corneal curvature and central corneal thickness (CCT) were measured using a Pentacam. SSI and biomechanical corrected IOP (bIOP) were measured *via* corneal visualization Scheimpflug technology (Corvis ST). The statistical analyses included one-sample Kolmogorov–Smirnov tests and normal distribution histogram methods, Levene variance homogeneity tests, Pearson’s correlation analyses, multiple linear stepwise regression analyses, one-way ANOVA, and LSD *t*-tests.

**Results:** The mean (±SD) age of the 240 participants was (24.97 ± 4.16) years. The SSI was positively correlated with spherical, cylinder, SE, CCT, IOP, and bIOP and negatively correlated with K1 and AL (*r* = 0.475, 0.371, 0.497, 0.169, 0.291, 0.144, −0.154, and −0.464, respectively; all *p* < 0.05), but were not correlated with age, K2, or Km (all *p* > 0.05). Multiple linear regression analysis performed with SSI as the dependent variable, and spherical, cylinder, K1, CCT, and IOP as independent variables produced the following regression equation: SSI = 0.989 + 0.017 spherical + 0.042 cylinder +0.018 IOP (*R* ^2^ = 0.402, *F* = 31.518, *p* < 0.001). The SSI values in the low-, moderate-, high-, and ultra-high myopia groups were 0.945 ± 0.135, 0.940 ± 0.128, 0.874 ± 0.110, and 0.771 ± 0.104, respectively. The values decreased sequentially, and the differences between pairs were statistically significant (all *p* < 0.05), except for that between the low- and moderate-myopia groups (*p* > 0.05).

**Conclusion:** SSI decreased with increasing myopia and astigmatism in the Chinese participants. The SSI was significantly lower in high and ultra-high myopia, especially ultra-high myopia. These findings indicate that increased corneal elasticity may be related to the pathogenesis of high and ultra-high myopia.

## Background

Myopia affected 22.9% of the world’s population in 2020 and is predicted to increase to 49.8% by 2050. Up to 9.8% of the world’s population is affected by high myopia (Holden et al., 2016), and the global burden of myopia is increasing annually (International Myopia Institute, et al., 2021). High myopia, especially ultra-high myopia, can cause biomechanical stretching and is associated with a series of complications, including myopic macular degeneration, glaucoma, cataract, and retinal detachment, which may increase the risk of blindness (Saw et al., 2005; Verkicharla et al., 2015) and may be attributed to the eyeball’s biomechanical properties (Tang et al., 2021). Thus, studying the biomechanical changes of the cornea is important for exploring myopia pathogenesis and prevention strategies.

The Corvis ST is a new biomechanical measurement instrument that integrates ultra-high speed Scheimpflug technology into an instrument that provides a non-contact measurement of intraocular pressure (IOP) and can dynamically record and analyze the biomechanical changes of corneal compression morphology and morphological reduction *in vivo* (Shi et al., 2018). The stress-strain index (SSI) is a new parameter provided by the Corvis ST. SSI maps can be used to estimate the regional variation of intrinsic elastic properties of the cornea (Zhang et al., 2021).

Recent studies have demonstrated the good repeatability of SSI in eyes of different CCT and in myopic eyes (Wang et al., 2021; Lu et al., 2022). However, SSI values may differ among races (Chua et al., 2017; Vinciguerra et al., 2022). Liu et al. reported that the SSI was relatively stable before 35 years of age and was positively correlated with anterior corneal curvature and IOP and negatively correlated with axial length (AL) in a healthy Chinese population (Liu et al., 2020). Thus, SSI could be suitable for the assessment of reduction in corneal stiffness in the natural progression of keratoconus and help recognize potential keratoconic eyes (Padmanabhan et al., 2022; Liu et al., 2021a). Some studies reported correlations between SSI values and IOP; therefore, SSI values may also be used for the differentiation of glaucoma types (Ye et al., 2021; Silva et al., 2022).

Two studies have reported SSI values in myopia (Han et al., 2020; Liu et al., 2021b). Han et al included patients with low and high myopia but did not include those with moderate or ultra-high myopia, which may be complicated with various conditions. Moreover, the relationships between SSI and spherical or cylinder were unclear. Liu et al. divided myopic eyes into groups with AL<26 mm and AL≥26 mm. In their study, the relationships between the basic parameters of myopia and SSI, such as spherical, cylinder, and spherical equivalent (SE), were unclear. Thus, the present study investigated the differences in corneal biomechanical parameter SSI among low, moderate, high, and ultra-high myopia to obtain SSI reference values for different degrees of myopia in a Chinese population. This study also analyzed the relevant factors of SSI.

## Methods

### Subjects

The cross-sectional study collected 240 right eyes from 240 myopic patients attending Henan Eye Hospital between January 2021 and February 2022. Written informed consent was obtained from each subject before any medical examination. This study followed the principles of the Declaration of Helsinki, and the study protocol was approved by the Ethics Committee of Henan Eye Hospital.

The inclusion criteria were patients 18–34 years of age with IOP ≤22 mmHg, diopter ≤ −0.50 D, cylinder > −5.00 D, and a stable diopter in the past 2 years (annual myopia deepening ≤0.5 D). The exclusion criteria were patients with a history of orthokeratology lens wearing, eye surgery, ocular trauma, keratoconus, corneal scar, glaucoma, other eye diseases, systemic connective tissue disease, or immune dysfunction.

**FIGURE 1:**
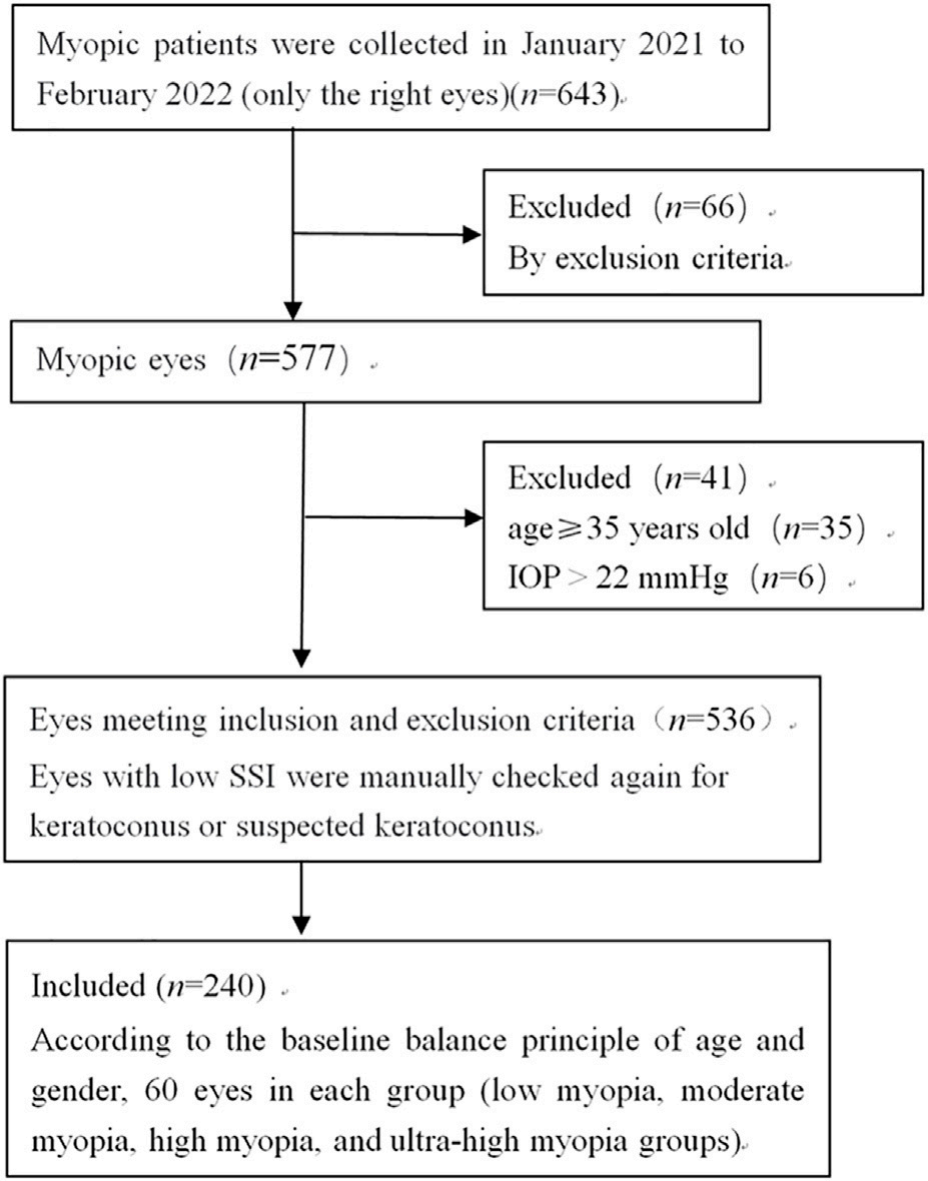
Flowchart of the study selection.

The participants were divided into four groups according to their SE: low myopia ( SE > −3.00 D to ≤ −0.50 D), moderate myopia (SE −6.00 D to −3.00 D), high myopia (SE > −10.00 D to < −6.00 D), and ultra-high myopia (SE ≤ −10.00 D) (Figure 1).

All subjects underwent complete ophthalmic examinations, including slit lamp microscopy, visual acuity measurements, optometry, IOP measurement, and fundus examination.

### SE, IOP, AL, Km, and CCT measurements

The sphere and cylinder were measured *via* computer automatic optometry and automatically integrated optometry, with SE = spherical +1/2 cylinder. The IOP was measured using a non-contact tonometer. AL was measured using an IOLMaster device. Corneal curvature and CCT were measured using a Pentacam. K1 and K2 were recorded, with Km calculated as (K1+K2)/2.

### Covris ST and SSI

SSI and bIOP were measured on a Covris ST instrument (Oculus, Germany). Each participant’s mandible was placed in the mandibular bracket, with their forehead pressed against the forehead bracket. Each participant was asked to open both eyes after blinking several times and to focus on the central red dot. The Corvis ST automatically identified the cornea apex after the pressure head was aligned. A uniform air pulse pressure was then applied to the cornea to obtain the dynamic parameters of the cornea biomechanics. The SSI and bIOP values were automatically generated by the Corvis ST. The examination was repeated five times, with an interval between measurements of 2–5 min. All examinations were performed by the same ophthalmologist between 9:00 and 17:00.

### Statistical analysis

Data were summarized into Microsoft Excel sheets, and the statistical analyses were performed using IBM SPSS Statistics for Windows, version 26.0 (IBM Corp., Armonk, NY, United States). One-sample Kolmogorov–Smirnov tests and the normal distribution histogram method were used to assess the normality of the distributions of the continuous variables. The Levene variance homogeneity tests was used to test the homogeneity of variance. Pearson’s correlation analysis was used to test the correlations between SSI and the other parameters. Multiple linear regression analysis was performed with SSI as the dependent variable and the other parameters as independent variables. The overall comparisons of age, spherical, cylinder, SE, K1, K2, Km, CCT, AL, IOP, bIOP, and SSI among the four groups were analyzed by one-way ANOVA, and LSD *t*-tests were used for post-hoc comparisons. *P* < 0.05 was considered statistically significant.

## Results

### Participant demographic and clinical characteristics

This study included a total of 240 patients (120 men, and 120 women, with 1:1 male-to-female ratios in each group). The SE ranged from −17.00 D to −0.75 D. The low, moderate, high, and ultra-high myopia groups, included 60 eyes each. The characteristics of the study participants are summarized in Table 1. The SSI ranged from 0.599 to 1.289, with a mean (±SD) value of 0.882 ± 0.138.

**TABLE 1:** Demographic and clinical characteristics of the study participants (*N* = 240).

Parameter	Mean ± SD	Range
Age (years)	24.97 ± 4.16	18–34
Spherical	−6.15 ± 3.47	−17.00–-0.75
Cylinder	−0.73 ± 0.75	−3.75–0.00
SE (D)	−6.51 ± 3.60	−17.00–-0.75
K1 (D)	43.58 ± 1.48	39.10–48.10
K2 (D)	42.62 ± 1.41	38.80–46.90
Km (D)	43.10 ± 1.36	38.95–47.10
CCT (μm)	538.32 ± 27.25	470.00–609.00
AL (mm)	26.11 ± 1.56	23.27–30.32
IOP (mmHg)	16.02 ± 2.55	10.3–22.0
bIOP	15.77 ± 2.28	10.5–26.7
SSI	0.882 ± 0.138	0.599–1.289

### Correlations between SSI and demographic or clinical characteristics

**FIGURE 2:**
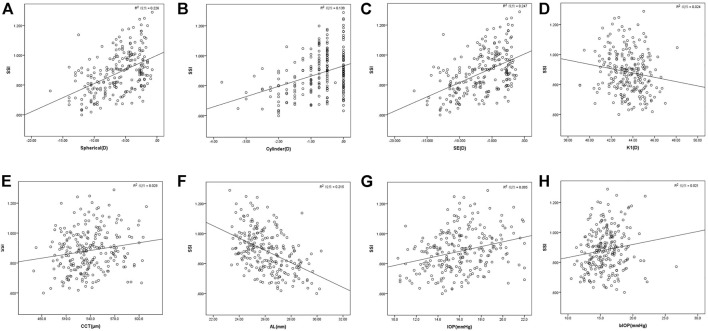
SSI is positively correlated with spherical **(A)**, cylinder **(B)**, SE **(C)**, CCT **(E)**, IOP **(G)**, and bIOP **(H)** and negatively correlated with K1 **(D)** and AL **(F)** (*r* = 0.475, 0.371, 0.497, 0.169, 0.291, 0.144, −0.154, and −0.464, respectively; all *p* < 0.05) (Pearson’s correlation analysis, *n* = 240).

Pearson’s correlation analysis showed that SSI was positively correlated with spherical, cylinder, SE, CCT, IOP, and bIOP and negatively correlated with K1 and AL (*r* = 0.475, 0.371, 0.497, 0.169, 0.291, 0.144, −0.154, and −0.464, respectively; all *p* < 0.05) but was not correlated with age, K2, or Km (all *p* > 0.05) (Table 2; Figure 2).

**TABLE 2:** Correlations between SSI and participant demographic or clinical characteristics (*N* = 240).

Parameter	SSI	
	*r*	*P*
Age (years)	−0.002	0.973
Spherical	0.475	<0.001*
Cylinder	0.371	<0.001*
SE (D)	0.497	<0.001*
K1 (D)	−0.154	0.017*
K2 (D)	0.028	0.668
Km (D)	−0.069	0.286
CCT (μm)	0.169	0.009*
AL (mm)	−0.464	<0.001*
IOP (mmHg)	0.291	<0.001*
bIOP	0.144	0.026*

(Pearson’s correlation analysis).

### Regression analysis of SSI and baseline characteristics

Pearson’s correlation analysis showed significantly prolonged AL with myopia deepening (*r* = −0.857, *p* < 0.001). Multiple linear regression analysis performed with SSI as the dependent variable, and spherical, cylinder, K1, CCT, and IOP as the independent variables produced the following regression equation: SSI = 0.989 + 0.017 spherical+0.042 cylinder +0.018 IOP (*R* ^2^ = 0.402, *F* = 31.518, *p* < 0.001) (Table 3).

**TABLE 3:** Multiple linear stepwise regression analysis with SSI as the dependent variable.

Variable	β	*t*	*P*	95% *CI*
Constant	0.989	3.806	<0.001*	0.505,1.536
Spherical	0.017	7.945	<0.001*	0.013,0.022
Cylinder	0.042	4.172	<0.001*	0.022,0.062
K1	−0.007	−1.514	0.131	−0.017,0.001
CCT	0.000	0.402	0.688	0.000,0.001
IOP	0.018	6.347	<0.001*	0.013,0.024

### Comparisons of demographic or clinical characteristics according to the degree of myopia

**FIGURE 3:**
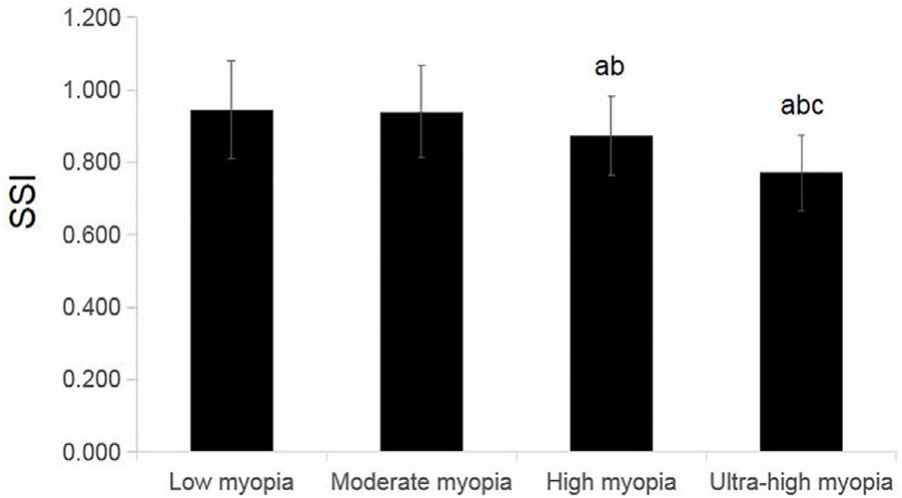
Comparisons of SSI for different degrees of myopia. Compared to the low-myopia group, ^a^ *P* < 0.05. Compared to the moderate-myopia group, ^b^ *P*<0.01. Compared to the high-myopia group, ^c^ *P*<0.01 (one-way ANOVA, LSD-*t* test, *n* = 60).

Age, K2, Km, CCT, and IOP did not differ significantly among the four groups (*F* = 0.082, *p* = 0.970; *F* = 0.754, *p* = 0.521; *F* = 2.092, *p* = 0.102; *F* = 2.360, *p* = 0.072; *F* = 0.980, *p* = 0.403). However, spherical, cylinder, SE, K1, AL, bIOP and SSI differed significantly among the four groups (*F* = 781.735, *p* < 0.001; *F* = 12.545, *p* < 0.001; *F* = 867.470, *p* < 0.001; *F* = 3.730, *p* = 0.012; *F* = 162.333, *p* < 0.001; *F* = 6.135, *p* < 0.001; *F* = 27.185, *p* < 0.001). Spherical, cylinder, and SE deepened, and AL elongated sequentially in the low-, moderate-, high-, and ultra-high myopia groups, with statistically significant differences in pairs (all *p* < 0.05). The SSI values in the low-, moderate-, high-, and ultra-high myopia groups decreased sequentially, with significant differences between pairs (all *p* < 0.05), except between the low and moderate myopia groups (*p* > 0.05) (Table 4; Figure 3).

**TABLE 4:** Comparisons of the demographic or clinical characteristics for different degrees of myopia.

Group	*n*	Age (years)	Spherical (D)	Cylinder (D)	SE (D)	K1 (D)	K2 (D)
Low myopia	60	24.75 ± 5.02	−2.17 ± 0.52	−0.38 ± 0.44	−2.36 ± 0.47	43.05 ± 1.49	42.42 ± 1.39
Moderate myopia	60	25.00 ± 3.68	−4.37 ± 0.86^a^	−0.58 ± 0.51	−4.66 ± 0.87^a^	43.76 ± 1.50^a^	42.80 ± 1.37
High myopia	60	25.12 ± 3.82	−7.02 ± 0.94^ab^	−0.81 ± 0.59^a^	−7.43 ± 0.99^ab^	43.64 ± 1.32^a^	42.57 ± 1.46
Ultra-high myopia	60	25.00 ± 4.01	−11.03 ± 1.60^abc^	−1.13 ± 1.08^abc^	−11.59 ± 1.55^abc^	43.85 ± 1.49^a^	42.66 ± 1.44
*F*		0.082	781.735	12.545	867.470	3.730	0.754
*P*		0.970	<0.001	<0.001	<0.001	0.012	0.521

Group	*n*	Km (D)	CCT (μm)	AL (mm)	IOP (mmHg)	bIOP (mmHg)	SSI
Low myopia	60	42.74 ± 1.37	542.83 ± 29.74	24.66 ± 0.71	15.55 ± 2.71	15.02 ± 2.19	0.945 ± 0.135
Moderate myopia	60	43.28 ± 1.37	538.33 ± 25.17	25.32 ± 0.80^a^	16.05 ± 2.76	15.61 ± 2.42	0.940 ± 0.128
High myopia	60	43.10 ± 1.34	541.32 ± 24.57	26.46 ± 0.94^ab^	16.26 ± 2.26	15.75 ± 2.07	0.874 ± 0.110^ab^
Ultra-high myopia	60	43.26 ± 1.33	530.78 ± 28.25	28.02 ± 1.09^abc^	16.22 ± 2.44	16.72 ± 2.16^abc^	0.771 ± 0.104^abc^
*F*		2.092	2.360	162.333	0.980	6.135	27.185
*P*		0.102	0.072	<0.001	0.403	<0.001	<0.001

(one-way ANOVA, LSD-*t*, test) Compared to the low myopia group, ^a^ *P* < 0.05. Compared to the moderate myopia group, ^b^ *P* < 0.05. Compared to the high myopia group, ^c^ *P* < 0.05.

## Discussion

SSI is a newly emerged biomechanical parameter that can be used to estimate the *in vivo* elastic properties of the cornea, following updates to the new Corvis ST instrument. The curves shift to the right and left for softer and stiffer corneas, respectively (Eliasy et al., 2019). The present study investigated the mean values and the differences in the corneal biomechanical parameter SSI among low, moderate, high, and ultra-high myopia. Overall, we found that the SSI value decreased significantly as myopia and astigmatism deepened in the myopic eyes of Chinese participants, especially those with high and ultra-high myopia.

Liu et al. reported that SSI was relatively stable before the age of 35, and then increased significantly with age in Chinese healthy population (Maklad et al., 2020). To eliminate the influence of age, patients under 35 years were selected as participants in our study, and this may be also one of the reasons that the SSI was not correlated with age in this study. In our study, the mean (±SD) SSI was 0.882 ± 0.138 in 18–34 years old Chinese myopic population, which was lower than 0.99 ± 0.17 in Chinese healthy eyes in 15–24 years old group, and 1.04 ± 0.15 in 25–34 years old group (Liu et al., 2020).

In this study, SSI was positively correlated with both spherical and cylinder (*r* = 0.475, 0.371). Few studies reported on the relationship between the SSI and spherical or cylinder.

To comprehensively consider the influence of SSI according to diopter, multiple linear regression analysis was performed after removing the co-linear factors (AL, SE, and bIOP) and with SSI as the dependent variable, and spherical, cylinder, K1, CCT, and IOP as independent variables. The following regression equation was obtained: SSI = 0.989 + 0.017 spherical +0.042 cylinder +0.018 IOP, which indicated that the SSI value was significantly affected by spherical, cylinder, and IOP in myopic eyes.

In this study, the SSI values decreased sequentially in the order of low, moderate, high, and ultra-high myopia. The changes in SSI in the different degrees of myopia groups were consistent with the regression analysis results mentioned above. The IOP did not differ significantly among the four groups, which indicated that the decrease in SSI in this study was mainly attributed to deepening myopia and astigmatism. The possible reasons for this finding include that eyes with lower SSI, which have larger corneal elasticity, are more likely to progress to high and ultra-high myopia; or the corneal elasticity increases in response to myopia and astigmatism deepening. Owing to the cross-sectional design of this study, whether a causal relationship exists between SSI and the occurrence and development of myopia and astigmatism, or whether lower SSI is a risk factor for the occurrence and development of myopia and astigmatism are unclear. Therefore, further longitudinal cohort studies or animal experiments are needed to further elucidate the relationship between the occurrence and development of myopia and SSI. Until now, no study has reported on the relationship between astigmatism and SSI. The specific mechanism requires further study.

Previous studies reported significant differences in SSI among frank keratoconus (0.67 ± 0.13), forme frusta keratoconus (0.77 ± 0.16), and normal eyes (0.83 ± 0.11) (*p* = 0.000), indicating an independent decrease in corneal stiffness in keratoconus eyes (Liu et al., 2021a). The SSI values in the low, moderate, and high groups in the present study were 0.945 ± 0.135, 0.940 ± 0.128, and 0.874 ± 0.110, respectively, which was close to the normal value in Chinese populations (Liu et al., 2021b). The SSI in the ultra-high myopia group was 0.771 ± 0.104, which was close to that in the forme frusta keratoconus group. The present study excluded eyes with keratoconus; thus, this phenomenon may be related to sampling error. However, this finding also indicated that regular checks and close follow-ups are needed for ultra-high myopic eyes, especially those with lower SSI values to allow the early detection and timely treatment of forme frusta keratoconus and frusta keratoconus.

## Limitations

The main limitation of this study was that it did not include normal eyes and lacked specific comparisons to eyes with normal diopter. Therefore, this topic requires further study. Moreover, as the population in this study was only 18–34 years of age, the characteristics of SSI in older myopia patients require further study.

## Conclusion

We obtained SSI reference values of eyes with different degrees of myopia in a Chinese population. We found that the SSI decreased significantly with deepening myopia and astigmatism. The SSI was significantly lower in the high and ultra-high myopia groups, especially in the ultra-high myopia group. The decreased SSI may be related to the pathogenesis of high and ultra-high myopia; however, the specific mechanism requires further study. Regular checks and close follow-ups are needed in ultra-high myopic eyes for the early detection and timely treatment of forme frusta keratoconus. A future long-term observational cohort study in children and adolescents with myopia is needed to further investigate the relationship between SSI and myopia development.

## Data Availability

The original contributions presented in the study are included in the article/Supplementary Material; further inquiries can be directed to the corresponding author.

## References

[B1] Chua J. Nongpiur M. E. Zhao W. Tham Y. C. Gupta P. Sabanayagam C. (2017). Comparison of corneal biomechanical properties between Indian and Chinese adults. Ophthalmology 124 (9), 1271–1279. 10.1016/j.ophtha.2017.03.055 28461014

[B2] Eliasy A. Chen K. J. Vinciguerra R. Lopes B. T. Abass A. Vinciguerra P. (2019). Determination of corneal biomechanical behavior *in-vivo* for healthy eyes using CorVis ST tonometry: Stress-strain index. Front. Bioeng. Biotechnol. 7, 105. 10.3389/fbioe.2019.00105 31157217 PMC6532432

[B3] International Myopia Institute Gao J. Liu K. Chen Z. (2021). IMI impact of myopia[J]. Chin. J. Exp. Ophthalmol. 39 (12), 1091–1103. 10.3760/cma.j.cn115989-20210623-00369

[B4] Han F. Li M. Wei P. Ma J. Jhanji V. Wang Y. (2020). Effect of biomechanical properties on myopia: A study of new corneal biomechanical parameters. BMC Ophthalmol. 20 (1), 459. 10.1186/s12886-020-01729-x 33213408 PMC7678063

[B5] Holden B. A. Fricke T. R. Wilson D. A. Jong M. Naidoo K. S. Sankaridurg P. (2016). Global prevalence of myopia and high myopia and temporal trends from 2000 through 2050. Ophthalmology 123 (5), 1036–1042. 10.1016/j.ophtha.2016.01.006 26875007

[B6] Liu G. Rong H. Pei R. Du B. Jin N. Wang D. (2020). Age distribution and associated factors of cornea biomechanical parameter stress-strain index in Chinese healthy population. BMC Ophthalmol. 20 (1), 436. 10.1186/s12886-020-01704-6 33143686 PMC7607623

[B7] Liu G. Rong H. Zhang P. Xue Y. Du B. Wang B. (2021b). The effect of axial length elongation on corneal biomechanical property. Front. Bioeng. Biotechnol. 9, 777239. 10.3389/fbioe.2021.777239 34926423 PMC8677453

[B8] Liu Y. Zhang Y. Chen Y. (2021a). Application of a scheimpflug-based biomechanical analyser and tomography in the early detection of subclinical keratoconus in Chinese patients. BMC Ophthalmol. 21 (1), 339. 10.1186/s12886-021-02102-2 34544392 PMC8454178

[B9] Lu N. J. Hafezi F. Rozema J. J. Hillen M. Hafezi N. Zhang J. (2022). Repeatability of a Scheimpflug tonometer to measure biomechanical parameters before and after myopic refractive surgery. J. Cataract. Refract. Surg. 48, 1057–1062. 10.1097/j.jcrs.0000000000000909 35171143

[B10] Maklad O. Eliasy A. Chen K. J. Wang J. Abass A. Lopes B. T. (2020). Fluid-structure interaction based algorithms for IOP and corneal material behavior. Front. Bioeng. Biotechnol. 8, 970. 10.3389/fbioe.2020.00970 32984273 PMC7483485

[B11] Padmanabhan P. Lopes B. T. Eliasy A. Abass A. Elsheikh A. (2022). *In vivo* biomechanical changes associated with keratoconus progression. Curr. Eye Res. 1-5, 982–986. 10.1080/02713683.2022.2058020 35385372

[B12] Saw S. M. Gazzard G. Shih-Yen E. C. Chua W. H. (2005). Myopia and associated pathological complications. Ophthalmic Physiol. Opt. 25 (5), 381–391. 10.1111/j.1475-1313.2005.00298.x 16101943

[B13] Shi Y. Huang X. D. Jiang Y. Q. (2018). Application of corneal visualization Scheimpflug technology in ophthalmology. Chin. J. Exp. Ophthalmol. 36 (6), 477–480. 10.3760/cma.j.issn.2095-0160.2018.06.016

[B14] Silva N. Ferreira A. Baptista P. M. Figueiredo A. Reis R. Sampaio I. (2022). Corneal biomechanics for ocular hypertension, primary open-angle glaucoma, and amyloidotic glaucoma: A comparative study by Corvis ST. Clin. Ophthalmol. 16, 71–83. 10.2147/OPTH.S350029 35035215 PMC8754459

[B15] Tang S. M. Zhang X. J. Yu M. Wang Y. M. Cheung C. Y. Kam K. W. (2021). Association of corneal biomechanics properties with myopia in a child and a parent cohort: Hong Kong children eye study. Diagn. (Basel) 11 (12), 2357. 10.3390/diagnostics11122357 PMC8700309 34943594

[B16] Verkicharla P. K. Ohno-Matsui K. Saw S. M. (2015). Current and predicted demographics of high myopia and an update of its associated pathological changes. Ophthalmic Physiol. Opt. 35 (5), 465–475. 10.1111/opo.12238 26303444

[B17] Vinciguerra R. Herber R. Wang Y. Zhang F. Zhou X. Bai J. (2022). Corneal biomechanics differences between Chinese and caucasian healthy subjects. Front. Med. 9, 834663. 10.3389/fmed.2022.834663 PMC8914014 35280913

[B18] Wang X. McAlinden C. Zhang H. Yan J. Wang D. Wei W. (2021). Assessment of corneal biomechanics, tonometry and pachymetry with the Corvis ST in myopia. Sci. Rep. 11 (1), 3041. 10.1038/s41598-020-80915-9 33542296 PMC7862660

[B19] Ye Y. Li Y. Zhu Z. Abu Said A. Z. M. Nguelemo Mayopa K. Akiti S. (2021). Effect of mydriasis-caused intraocular pressure changes on corneal biomechanical metrics. Front. Bioeng. Biotechnol. 9, 751628. 10.3389/fbioe.2021.751628 34900957 PMC8664602

[B20] Zhang H. Eliasy A. Lopes B. Abass A. Vinciguerra R. Vinciguerra P. (2021). Stress-strain index map: A new way to represent corneal material stiffness. Front. Bioeng. Biotechnol. 9, 640434. 10.3389/fbioe.2021.640434 33777912 PMC7991572

